# Study on the Mechanism of GABA-Rich Adzuki Bean Regulating Blood Glucose Based on the IRS/PI3K/AKT Pathway

**DOI:** 10.3390/foods13172791

**Published:** 2024-09-02

**Authors:** Xiujie Jiang, Ying Li, Zhenzhen Cao, Qingpeng Xu, Jiayu Zhang, Dongmei Cao, Xiaoxing Chi, Dongjie Zhang

**Affiliations:** 1 College of Food Science, Heilongjiang Bayi Agricultural University, Daqing 163319, China; jiangxiujie-2008@163.com (X.J.);; 2 Supervision, Inspection and Testing Center for Agricultural Products and Processed Products, Ministry of Agriculture and Rural Affairs, Daqing 163319, China; 3 National Coarse Cereals Engineering Research Center, Heilongjiang Bayi Agricultural University, Daqing 163319, China

**Keywords:** adzuki bean (*Vigna angularis*), GABA, T2DM, IRS/PI3K/AKT signaling pathway

## Abstract

The adzuki bean is a mature seed of the red bean leguminous plant, and people like to eat it because of its nutritious properties and moderate proportion of amino acids. Adzuki bean germination and the enrichment of GABA greatly improve the health effects of the adzuki bean. The effects of the GABA-rich adzuki bean on the expression of insulin-pathway-related genes and proteins in the liver of T2DM mice were studied via Western blotting and qPCR. The results showed that a GABA-rich adzuki bean diet could promote glycogen synthesis in the liver of T2DM mice, inhibit the activities of PEPCK and G-6-Pase, and significantly down-regulate the gene expression levels of *PEPCK*, *G6PC* and *FOXO1* (*p* < 0.05) and the phosphorylation levels of FOXO1 and GSK3β. In addition, it can also up-regulate the expression of the *AMPKα* gene and down-regulate the expression of the *SREBP1c* gene to inhibit the synthesis of triglycerides and cholesterol in T2DM mice. Lipid accumulation in mice can alleviate glucose and lipid metabolism disorders and play an effective role in regulating blood glucose at liver tissue targets. This study suggested that the GABA-rich adzuki bean can improve hyperglycemia in type 2 diabetic mice by activating the IRS/PI3K/AKT signaling pathway in the liver.

## 1. Introduction

In recent years, the effect of dietary nutrition on human chronic diseases has become a research hotspot in the food science community. Type 2 diabetes mellitus (T2DM) is a progressive metabolic disease and the most serious public health problem in the world [1]. More and more studies have shown that a bean-based diet is helpful in preventing and controlling the occurrence of T2DM, and the adzuki bean is one of the high-protein and low-calorie beans that people like to eat. It is rich in dietary fiber and resistant starch, which does not cause a significant burden in terms of postprandial blood sugar, and it also contains iron, calcium, phosphorus, and other trace elements for human health [2]. It is known as “the red pearl in grain beans” [3]. At the same time, the adzuki bean also contains substances with blood glucose reaction activity, which have a good inhibitory ability with respect to α-glucosidase and a positive impact on postprandial blood glucose reactions. It is one of the best beans for diabetic patients [4]. However, the extent of the adzuki bean’s contribution to the prevention and management of type 2 diabetes still requires further study [5], and beans have poor processing performance, rough/poor taste, are difficult to digest, and contain anti-nutritional factors, which limits their consumption as a staple food.

Germination is a simple and effective technology with which to improve the palatability, digestibility, and nutrient value of plants. During the germination of legumes, the reactivation of the seed’s metabolism after water absorption changes the nutritional, physiological, and biochemical characteristics of legumes, resulting in the catabolism of macronutrients and anti-nutritional compounds and the biosynthesis of secondary metabolites, which has potential health benefits [6]. Simultaneous germination can significantly increase the content of γ-aminobutyric acid (GABA) in legumes [7]. GABA branch synthesis is the main means by which to enrich GABA in grain and legume crops; that is, GABA is synthesized through the irreversible decarboxylation of Glu at the α-position under the catalysis of glutamate decarboxylase (GAD) [8]. During germination, protease and related rate-limiting enzymes such as GAD and DAO are activated in plant seeds and the protein begins to hydrolyze, which increases the content of the L-Glu substrate, thus providing sufficient conditions for GABA synthesis [9]. GABA is a non-protein amino acid that can induce alpha-cell to beta-cell transformations and increase insulin sensitivity. GABA can improve islet cell function, blood glucose homeostasis, and the autoimmune function in diabetic patients [10]. In addition, GABA enhances islet cell function by generating membrane depolarization and Ca^2+^ inflow, activating PI3K/Akt signaling factors and improving beta cell quality [11], and it is a new bioactive ingredient in food. The glutamate in the adzuki bean, which is present in high levels, is the precursor for GABA synthesis, and it has the potential to enrich GABA by means of plant metabolism, which greatly improves the health effects of the adzuki bean. GABA-rich foods have been a hot spot in functional food research for a while, but the pathway and mechanism by which GABA-rich products regulate blood glucose need to be further explored.

In this paper, we studied the distribution and changes in hepatic glycogen in T2DM mice via PAS staining and quantitative analysis; we studied the expression of insulin-pathway-related genes and proteins via Western blotting and q-PCR molecular techniques to reveal the regulation pathway and molecular mechanism of hepatic glycogen metabolism in T2DM mice. Our hope is to provide theoretical support for the regulation of blood sugar through the use of natural foods.

## 2. Materials and Methods

### 2.1. Materials

Adzuki bean (*Vigna angularis*) raw materials (variety: Pearl Red) were purchased from Daqing Ruizefeng Technology Co., Ltd., Daqing, Heilongjiang Province, China. GABA (HPLC ≥ 99%) was purchased from Shanghai Yuanye Biotechnology Co., Ltd., Shanghai, China. GSK3, p-GSK3β, AMPKα, IRS1, AKT, p-AKT, FOXO1, and p-FOXO1 antibodies were purchased from Chengdu Zhengneng Biotechnology Co., Ltd., Chengdu, Sichuan Province, China. Other biochemical reagents and enzyme activity detection kits, such as G-6-PasePEPCK, were purchased from Nanjing Jiancheng Biotechnology Co., Ltd., Nanjing, Jiangsu Province, China. The experimental animals were purchased from Changchun Yisi Experimental Animal Technology Co., Ltd., Changchun, China (License No.: SCXK (Ji) 2018-0007).

### 2.2. Methods of Adzuki Bean Enrichment

Appropriate amounts of the adzuki bean were washed and soaked in a 0.7% sodium hypochlorite solution for 15 min. The adzuki bean and soaking solution were treated according to a 1:5 ratio, and the soaking conditions were as follows: soaking time of 16 h, soaking temperature of 35 °C, and pH of 5. After soaking, the substance was evenly spread on a plate, covered with four layers of gauze, and then put into a vacuum incubator for 16 h. The total germination time was 48 h, and a concentration of sodium glutamate (MSG) was sprayed every 1 h during germination. The germination temperature was 35 °C. Finally, we obtained the GABA-rich germinated adzuki bean products [12].

### 2.3. Animal Modeling and Grouping

SPF C57BL/6J male mice (n = 74) (6 weeks old; body weight: 18~22 g): A T2DM mice model group was fed TP23300 60% high-fat feed for 4 weeks. STZ (50 mg/kg; 0.1 mol/L citric acid buffer pH = 4.0) was injected intraperitoneally after prohibiting the intake of food for 12 h in the model group. The fasting blood glucose of mice was measured on the 3rd day after injection. When the fasting blood glucose of mice was more than 12 mmol/L for 3 days, the model was established successfully [13]. The successful mice were divided into the following groups: model group (M); HFD + low-dose GABA-rich adzuki bean group (TF1, 15 g/100 g); HFD + medium-dose GABA-rich adzuki bean group (TF2, 25 g/100 g); HFD + GABA-rich adzuki bean group (TF3, 35 g/100 g); adzuki bean intervention group (B, 35 g/100 g); HFD + GABA intervention group (TG, 0.1 g/kg); and HFD + metformin group (TS, 0.1 g/kg). Eight mice in each group were fed the relevant diet and treated with drugs for 6 weeks. After dietary intervention, the blood and organs of the mice in each treatment group were packed and stored in a −80 °C refrigerator for later use.

### 2.4. Detection of Liver Glycogen Content

Liver glycogen was detected via the method by Purwana et al. [14]. First, we weighed 50 mg of liver sample in a test tube, added 3 times the volume of alkali solution to the sample, and bathed the sample in water at 100 °C for 20 min until the hydrolysis was completed; then, we cooled the sample with running water. The obtained glycogen hydrolyzate was diluted into a 1% glycogen working solution, and a certain amount of distilled water was added. We diluted the glycogen working solution 10 times again, put 100 μL of each dilution into a 1.5 mL centrifuge tube, added 200 μL of anthrone chromogen, shook and mixed the solution, placed the tubes in a water bath at 100 °C for 5 min again, waited until it was cool, and measured the solution’s absorbance at a wavelength of 570 nm. The blank control was distilled water and the solution in the tube was liquid. The content was calculated according to the following formula:

glycogen content = (measuring tube OD − standard tube OD) × standard tube content × dilution of multiple solution samples ×measured dilution multiple/1.11.

### 2.5. PAS Staining of Liver Glycogen

According to the method by Choat et al. [15], for PAS staining, an appropriate amount of liver samples was taken and fixed in Carnoy’s solution at 4 °C for 6 h. After gradient dehydration with ethanol and paraffin embedding, 5 μm tissue sections were prepared. They were treated with periodate for 15 min, rinsed with Schevert for 30 min, rinsed for 5 min, and treated with hematoxylin for 5 min. After the rinsing and bluing treatment, dehydrated sealing, microscope observations, and photo retention followed.

### 2.6. Determination of the Content of Key Enzymes in Glucose Metabolism

We took an appropriate amount of liver tissue, added the extract at a ratio of 1:10, and homogenized the sample tissue in an ice bath environment. It was centrifuged at a low temperature for 10 min (8000× *g*). We removed the supernatant and put it on ice for inspection. Phosphoenolpyruvate carboxykinase (PEPCK) was determined according to the kit’s instructions, and glucose-6-phosphatase (G-6-Pase) was determined according to ELISA kit methods.

### 2.7. Liver Tissue Immunofluorescence

Liver tissue samples from groups C, M, TF3, and TS were selected for immunofluorescence staining. The detection of GSK3β, p-AKT, and PI3K was completed by Wuhan Bolf Biotechnology Co., Ltd. (Wuhan, China).

### 2.8. Determination of Liver-Related Gene Expression

An appropriate amount of liver tissue was taken and ground into a powder via liquid nitrogen pre-cooling treatment under aseptic conditions; then, 1 mL of Trizol lysate was added, and the operation was carried out according to the instructions of the total RNA extraction kit. The extract was dissolved in DEPC water, and the RNA OD 260/280 value was detected in the range of 1.8~2.1. The RNA concentration was recorded and then the extract was sealed and stored at −80 °C for later use. cDNA was prepared according to the Prime Script TM RT kit, and then real-time fluorescent quantitative PCR was performed. The primers involved are shown in Table 1.

### 2.9. Western Blot Analysis

Western blot technology was used to detect the gene protein expression of p-GSK3β, GSK3β, IRS1, PI3K, AKT, p-AKT, AMPKα, FOXO1, and p-FOXO1 in the liver tissues of mice in the C, M, TF3, and TS groups. For the specific methods, refer to Han. X. With minor modification, each 100 mg mouse liver sample was added to 500 µL of lysate-containing phosphatase inhibitor and quickly ground into powder on ice. This powder was subjected to a static reaction for 30 min and centrifuged at a low temperature at 1.2 × 10^4^× *g* for 15 min. The protein concentration of the above extraction solution was detected via the BCA protein kit method. Then, DS-PAGE electrophoresis, membrane transfer, and immune reactions were performed, and finally, development and analysis were performed [16].

### 2.10. Data Statistics and Analysis

The presented biochemical indexes of hypoglycemia in animals are the mean ± standard deviation (n ≥ 6). A one-way analysis of variance with SPSS 22.0 software and Duncan’s multiple comparison method were used for statistical analysis of the data. *p* < 0.05 indicated statistical significance between groups or within groups. GraphPad prism 7.04 was used to draw straight lines and column graphs, and we used PowerPoint 2010 to combine individual graphs.

## 3. Results

### 3.1. Effects of Different Treatments on Blood Glucose and Blood Lipid Levels in T2DM Mice

**Figure 1:**
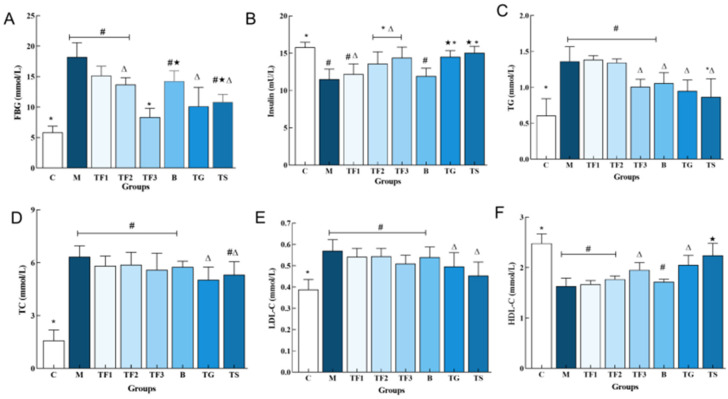
Effect of GABA-rich adzuki beans on the serum glucose and lipid metabolism indexes in T2DM mice: (**A**) serum FBG; (**B**) serum INS; (**C**) TG content in serum; (**D**) TC content in serum; (**E**) LDL-CTC content in serum; and (**F**) HDL-C TC content in serum. The values are presented as the mean ± SEM (*n* = 6). Different characters indicate significant differences between groups (*p* < 0.05). Abbreviations in the picture: normal control (C); model group (M); HFD + low-dose GABA-rich adzuki bean group (TF1); HFD + medium-dose GABA-rich adzuki bean group (TF2): HFD + GABA-rich adzuki bean (TF3); adzuki bean intervention group (B); HFD + GABA intervention group (TG); and HFD + metformin group (TS).

Abnormal serum biochemical indexes are closely linked to T2DM, of which blood glucose and insulin are important markers of glucose metabolism and islet function. As can be seen in Figure 1A, the FBG (18.21 ± 2.69 mmol/L) of mice in the M model group was significantly higher than that in the C group (*p* < 0.05). The level of FBG in the TF3 group decreased significantly (*p* < 0.05) compared with that in the GABA (TG) and metformin (TS) positive control group, by 21.05% and 29.66%, respectively. The results showed that the GABA-rich adzuki bean had a certain effect on blood glucose, and the change in blood glucose was related to intake. Figure 1B compares the serum insulin levels of mice in different groups. The INS content of mice in the M model group was significantly lower than that of mice in the C group (*p* < 0.05), indicating the insulin deficiency of mice in the M group. In addition, type 2 diabetes mellitus is often accompanied by lipid metabolism disorder; the serum lipid factors of T2DM mice in different dietary intervention groups were detected (Figure 1C–F). Compared with the control group, the levels of TG, TC, and LDL-C in the M model group were significantly increased (*p* < 0.05, Figure 1C,D) and the levels of HDL-C were significantly increased (*p* < 0.05, Figure 1F). It was revealed that the diabetic mice, whose condition was induced by a high-fat diet combined with STZ, had severe dyslipidemia. After 6 weeks of GABA-rich adzuki bean and drug intervention, the levels of TG, TC, and LDL-C in the TG and TS groups were significantly increased, and the levels of HDL-C in the TG group were not significantly different (*p* > 0.05).

### 3.2. Effects of Different Treatment Groups on Liver Glycogen Synthesis in T2DM Mice

**Figure 2:**
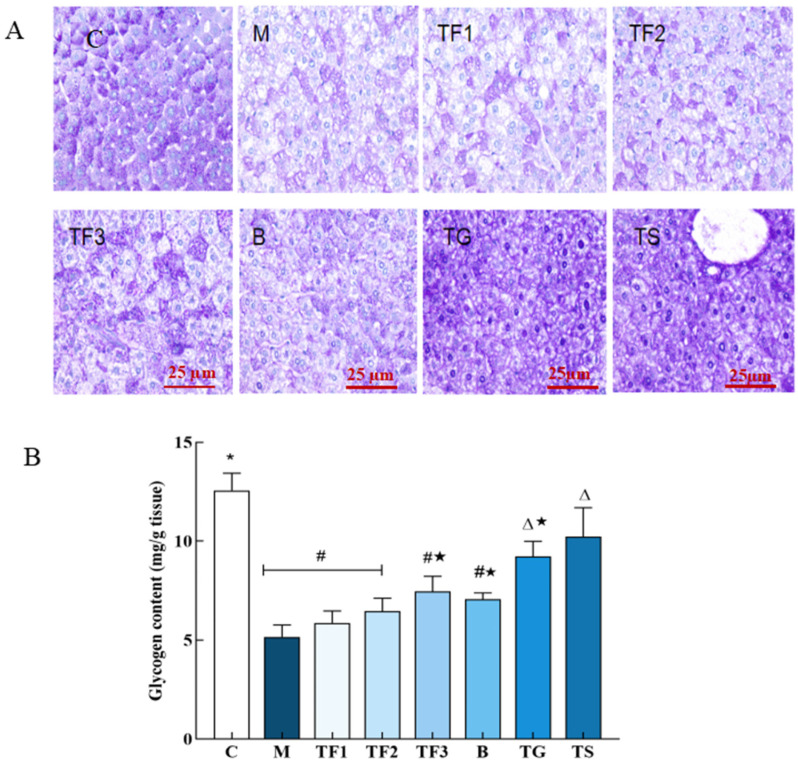
Effects of different treatments on PAS staining and glycogen content in mouse liver tissue. (**A**) PAS staining of glycogen in liver of mice in different groups (×400); (**B**) glycogen content in liver tissue of mice in different groups; different characters indicate that there were significant differences among the data groups (*p* < 0.05). Abbreviations in the picture: normal control (C); model group (M); HFD + low-dose GABA-rich adzuki bean group (TF1); HFD + medium-dose GABA-rich adzuki bean group (TF2): HFD + GABA-rich adzuki bean (TF3); adzuki bean intervention group (B); HFD + GABA intervention group (TG); and HFD + metformin group (TS).

In this experiment, the storage and distribution of liver glycogen in mice in different treatment groups were studied via PAS staining and quantitative analysis. The results are shown in Figure 2.

After PAS staining, the liver glycogen of the normal control group C showed deep purple staining; the amount of liver glycogen in the M model group was obvious, and there were more fat vacuoles. After 6 weeks of intervention with the different doses of GABA-rich adzuki beans and metformin treatment, it was found that the liver glycogen staining degree of TF1, TF2, and TF3 treatment increased with the dose, and the effect in the high-dose TF3 group was second only to the TG and TS positive control (Figure 2A). The quantitative results of glycogen content and the PAS staining results were complimentary. From Figure 2B, it can be seen that the glycogen content of T2DM mice in the M model group is significantly lower than that in the C control group. Different doses of GABA-rich adzuki beans and drug treatment can improve the liver glycogen content in mice to different degrees. The ability of TF3, TG, and TS groups to recover glycogen synthesis in T2DM mice may promote liver glycogen synthesis in T2DM mice by alleviating liver lipid peroxidation and histological damage.

### 3.3. Effects of Different Treatment Groups on Glycogen Synthase Kinase 3 β in T2DM Mice

**Figure 3:**
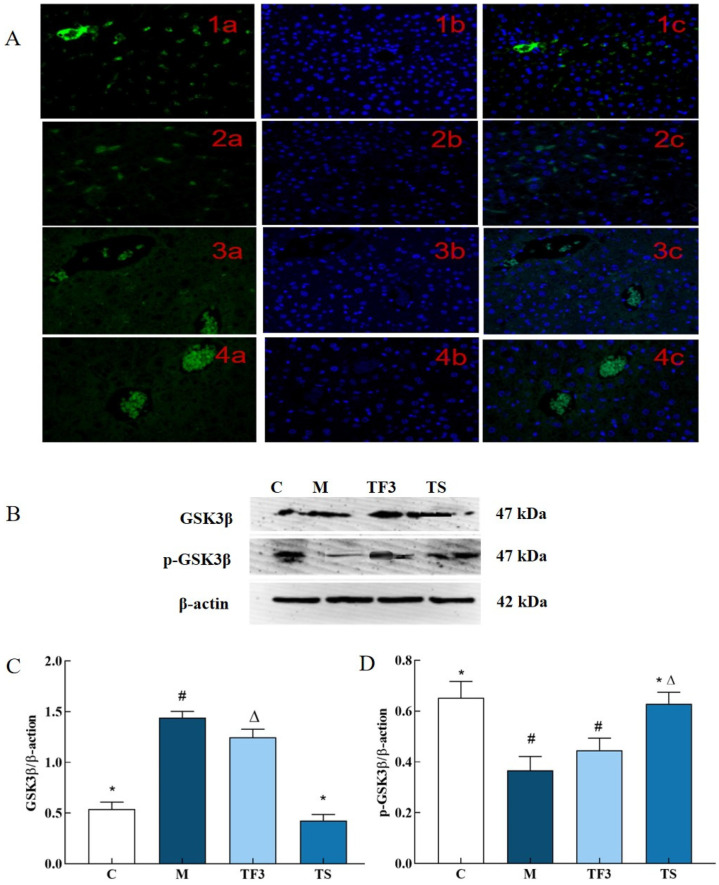
Effects of different treatment groups on protein expression of hepatic GSK3β in T2DM mice. (**A**) 1, normal C group; 2, M model group; 3, TF3 group; and 4, TS group, in which a—FITC marker, b—DAPI restaining nucleus and c—merge; (**B**) the protein bands of GSK3β and its phosphorylated p-GSK3β; (**C**) shows the expression level of GSK3β protein in different groups of mice; and (**D**) shows the expression level of p-GSK3β phosphorylation. There were significant differences among the groups represented by different characters (*p* < 0.05). Abbreviations in the picture: normal control (C), model group (M), HFD + GABA-rich adzuki bean (TF3), and HFD + metformin group (TS).

In this study, the representative experimental groups of C, M, TF3, and TS were selected to evaluate the expression status of GSK3β in mouse liver cells via immunofluorescence and the expression levels of p-GSK3β and GSK3β protein via Western blotting. The results are shown in Figure 3.

As shown in Figure 3A, which shows the fluorescence microscopy results, the expression of GSK3β in mouse liver cells produced green fluorescence. GSK3β produced stronger fluorescence in C control cells, while the M model group showed a weaker fluorescence intensity. Compared with the C group, the green, fluorescent dots of hepatocytes in the M model group were evidently sparse and darkened, which indicated that the expression level of GSK3β was significant (*p* < 0.05). The expression level of GSK3β in hepatic cells increased to different degrees after treatment with a high dose of GABA-rich adzuki bean (TF3) and metformin (TS), and TF3 and TS were statistically significant compared with M (*p* < 0.05). 

As can be seen in Figure 3B–D, the expression level of GSK3β total protein in the liver tissue of mice in the M model group was significantly up-regulated compared with the normal control (*p* < 0.05); i.e., it was 2.67 times higher than that of the normal control. At the same time, TF3 and TS can significantly alleviate the phenomenon of high GSK3β protein levels caused by hyperglycemia and significantly down-regulate the expression level of the GSK3β protein in T2DM mice livers, but the expression level of GSK3β total protein in the TF3 group is still at a higher level than that in the C and TS groups. The effects of TF3 and TS on GSK3β in the liver of T2DM mice were consistent at both the protein and cellular level. In addition, TF3 dietary intervention significantly increased the phosphorylation level of GSK3β, which led to the dephosphorylation of GSK3β induced by diabetes.

### 3.4. Effects of Different Treatments on Gluconeogenesis Rate-Limiting Enzyme Activity in T2DM Mice

**Figure 4:**
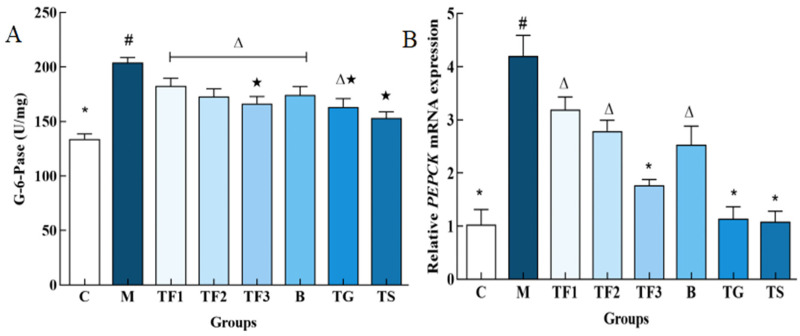
Effects of different treatment groups on gluconeogenesis rate-limiting enzyme activity in T2DM mice. (**A**) Shows the G-6-Pase enzyme activity map and (**B**) shows the PEPCK enzyme activity map with different characters indicating significant differences between groups (*p* < 0.05). Abbreviations in the picture: normal control (C); model group (M); HFD + low-dose GABA-rich adzuki bean group (TF1); HFD + medium-dose GABA-rich adzuki bean group (TF2): HFD + GABA-rich adzuki bean (TF3); adzuki bean intervention group (B); HFD + GABA intervention group (TG); and HFD + metformin group (TS).

It can be seen from Figure 4 that the activities of G-6-Pase and PEPCK in the liver of the M model group are significantly higher than those of the normal control group C and other dietary and drug treatment groups (*p* < 0.05). The activities of G-6-Pase and PEPCK in the M group are 1.52 times higher than those in the C group and 2.31 times higher than those in the C group. The activities of PEPCK in TF3, TG, and TS groups are 41.5%, 40.02%, and 46.16%, respectively, and there were no significant differences among the three groups (*p* > 0.05). The results further prove that a GABA-rich adzuki bean diet could inhibit glycogen decomposition, the gluconeogenesis rate-limiting enzyme G-6-Pase, and PEPCK activities in T2DM mice, suggesting that it promotes the glycolysis pathway in reverse and then regulates blood glucose concentration in T2DM mice and alleviates hyperglycemia symptoms in mice.

### 3.5. Effects of Different Treatments on the Expression of Glucose-Metabolism-Related Genes in the Liver of T2DM Mice

**Figure 5:**
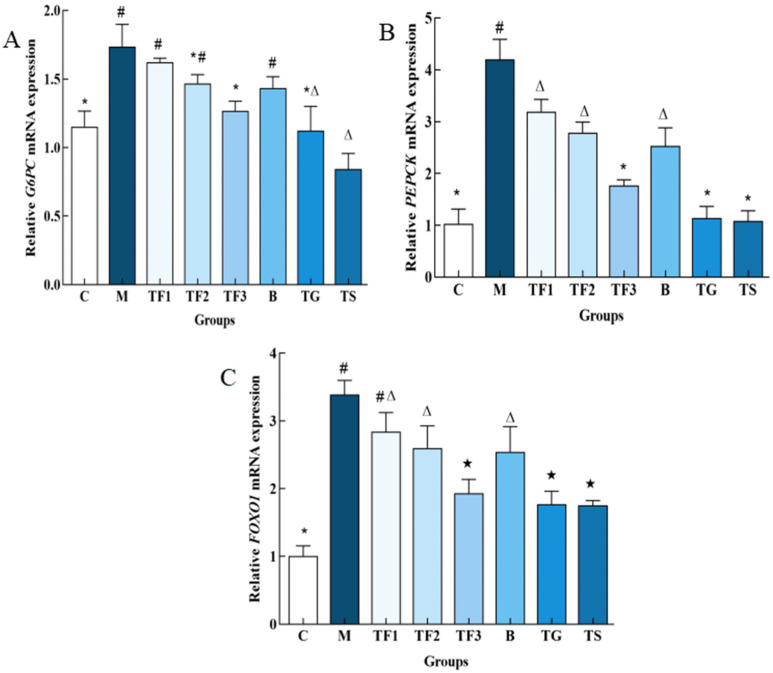
Effects of different treatments on the expression of glucose metabolism-related genes in mice. (**A**) Shows the relative expression level of the *G6PC* gene; (**B**) shows the relative expression level of the *PEPCK* gene; and (**C**) shows *FOXO1*. There are significant differences among different characters in the figure (*p* < 0.05). Abbreviations in the picture: normal control (C); model group (M); HFD + low-dose GABA-rich adzuki bean group (TF1); HFD + medium-dose GABA-rich adzuki bean group (TF2): HFD + GABA-rich adzuki bean (TF3); adzuki bean intervention group (B); HFD + GABA intervention group (TG); and HFD + metformin group (TS).

The effects of the GABA-rich adzuki bean diet on *PEPCK* and *G6PC* gene expression in the liver of T2DM mice, determined via the qPCR technique at the molecular level, are shown in Figure 5A. The expression of the *G6PC* gene in the liver of the M model group was up-regulated significantly compared with that of the C group (*p* < 0.05), and the expression of the *G6PC* gene in the liver of the TF3 group was down-regulated by 27.16% compared with the M group, and up-regulated by 33.33% compared with the C control group. With respect to the *PEPCK* gene, different doses of the GABA-rich adzuki bean and drug treatment can significantly inhibit the expression level of the *PEPCK* gene. The inhibitory effect of TF1, TF2, and TF3 dietary intervention on PEPCK is dose-dependent, while the inhibitory effect of TGTS in the high-dose TF3 group is equivalent to that of the drug treatment, and there is no significant difference among the three groups (*p* > 0.05, Figure 5B). In Figure 5C, we can see the expression level of the *FOXO1* gene in the M model group. TF3 diet and drug intervention can significantly down-regulate the expression level of the *FOXO1* gene in the liver of T2DM mice and also increase the protein phosphorylation level of *FOXO1*.

### 3.6. Effects of Different Treatments on the Expression of Genes Related to Liver Lipid Metabolism in T2DM Mice

**Figure 6:**
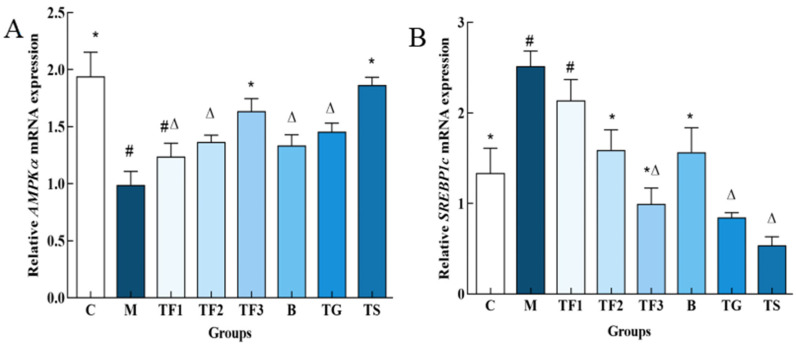
Effects of different groups on *AMPK α* and *SREBP1c* gene expression in the liver of mice. (**A**,**B**) indicate *AMPKα* and *SREBP1c* gene expression levels, respectively, and different characters indicate significant differences between groups (*p* < 0.05). Abbreviations in the picture: normal control (C); model group (M); HFD + low-dose GABA-rich adzuki bean group (TF1); HFD + medium-dose GABA-rich adzuki bean group (TF2): HFD + GABA-rich adzuki bean (TF3); adzuki bean intervention group (B); HFD + GABA intervention group (TG); and HFD + metformin group (TS).

In this study, the expression levels of *AMPKα* and *SREBP1c* genes in the liver of T2DM mice with different doses of dietary intervention were detected. The results are shown in Figure 6.

As shown in Figure 6A, the expression level of the *AMPKα* gene in the liver of mice in the M model group was significantly lower than that in the C group (*p* < 0.05). The medium- and high-dose GABA-rich adzuki bean groups and metformin groups exhibited significant up-regulation of the expression level of *AMPKα* in T2DM mice, among which the TF3 high-dose group exhibited an increased expression level of the *AMPKα* gene by 2.09-fold. There were no significant differences compared with the normal TG and C groups (*p* > 0.05). Figure 6B shows that the expression level of the SREBP1c gene in the M model group is significantly higher than that in the C group and the medium- and high-dose diet group. The TF2, TF3, B, TG, and TS groups exhibited a significant down-regulation in the expression level of the *SREBP1c* gene in the liver of T2DM mice (*p* < 0.05), but there were no significant differences between TF3, TG, and TS (*p* > 0.05).

### 3.7. Effects of Different Treatment Groups on the Expression of Insulin Pathway-Related Proteins in T2DM Mice

**Figure 7:**
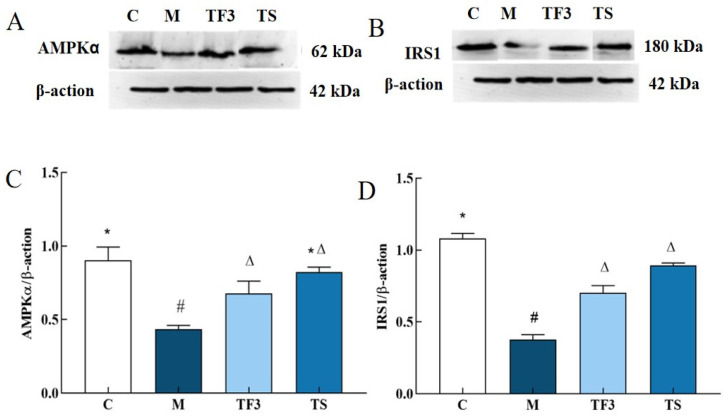
Effects of different groups on AMPKα and IRS1 protein expression in liver of mice. (**A**) AMPK α protein band; (**B**) IRS1 protein band; (**C**) AMPK α protein expression level; (**D**) IRS1 protein expression level; different characters indicate significant differences between groups (*p* < 0.05). Abbreviations in the picture: normal control (C); model group (M); HFD + GABA-rich adzuki bean (TF3); and HFD + metformin group (TS).

The expression of the insulin pathway factor AMPKα and the IRS 1 protein is shown in Figure 7. Compared with the normal group, the expression of AMPK α and the IRS proteins in the liver of the M model group were significantly down-regulated. After 6 weeks of dietary and drug intervention, the expression of the AMPKα and IRS proteins in the liver of the TF3 and TS groups were significantly up-regulated compared with those of the M model group (*p* < 0.05), and there were no significant differences between the two groups (*p* > 0.05).

**Figure 8:**
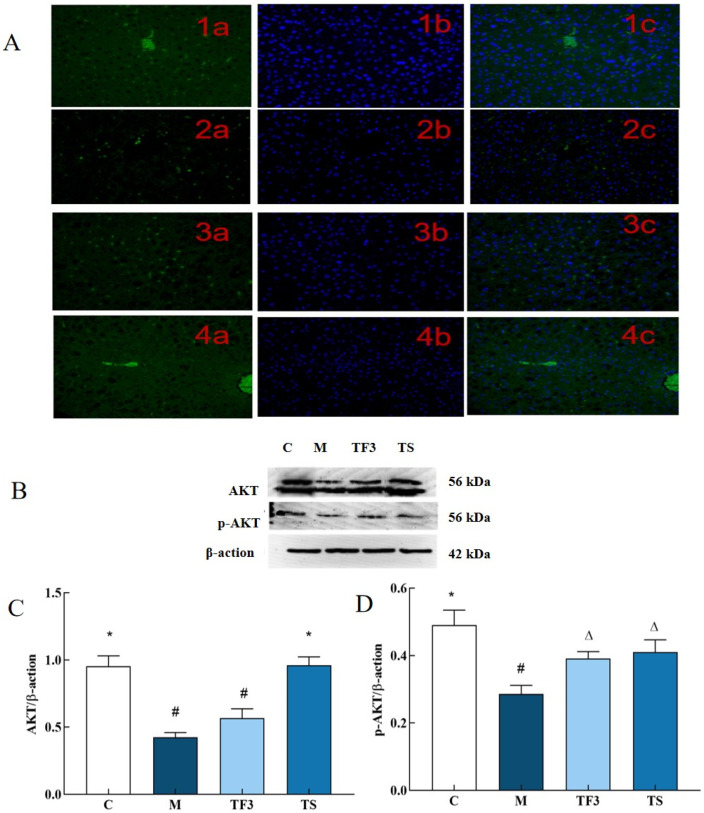
Effects of different groups on the expression of the AKT and p-AKT protein in the liver of mice. (**A**) 1 stands for C group; 2 stands for M model group; 3 stands for TF3, high-dose adzuki bean group rich in GABA; and 4 stands for TS metformin group, in which a was FITC labeled, b was DAPI restained nucleus, and c was merged. (**B**) Shows the protein bands of AKT and its phosphorylated p-AKT. (**C**) Shows the expression level of the AKT protein in different groups of mice. (**D**) Shows the phosphorylation expression level of the p-AKT protein. There were significant differences among the groups represented by different characters (*p* < 0.05). Abbreviations in the picture: normal control (C); model group (M); HFD + GABA-rich adzuki bean (TF3); and HFD + metformin group (TS).

As can be seen from Figure 8A, compared with the normal control group C, p-AKT phosphorylation in the liver cells of mice in the M model group showed a low expression, and the green, fluorescent dots are few and dim. On the other hand, TF3 and TS treatments significantly restored the fluorescence intensity of the liver cells in T2DM mice, and the number and intensity of the green, fluorescent dots in the liver cells in the M model group underwent significant changes. These results suggest that TF3 and TS may up-regulate the phosphorylation of the p-AKT protein in liver cells. In order to verify the immunofluorescence results, Western blotting was used to further analyze the expression and phosphorylation of the AKT protein, as shown in Figure 8B–D. As shown in Figure 8C,D, the expression of the total AKT protein in the liver tissues of mice in the M model group was significantly decreased compared with that of the normal control group (*p* < 0.05). After the intervention of a high dose of GABA-rich adzuki bean (TF3), the total AKT protein expression level and the phosphorylation degree of AKT in the liver of T2DM mice were significantly increased (*p* < 0.05), and the phosphorylation of AKT in the liver cells of T2DM mice was also restored. The effect was not significantly different from that of metformin.

**Figure 9:**
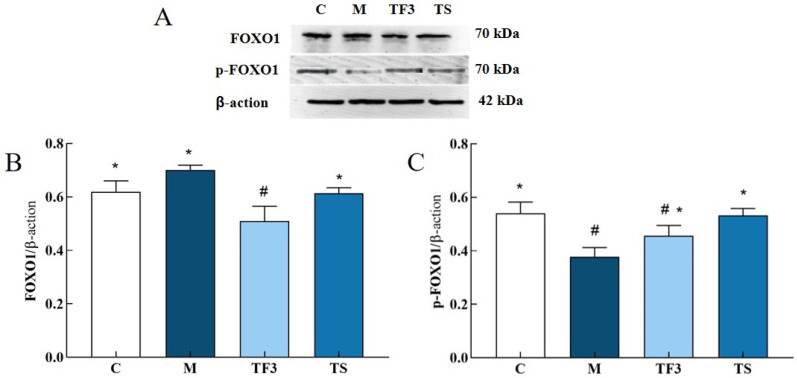
Effects of different groups on FOXO1 and p-FOXO1 protein expression in the liver of T2DM mice. (**A**) Shows the protein bands of FOXO1 and p-FOXO1, (**B**) shows the expression level of the FOXO1 protein in different groups of mice, and (**C**) shows the phosphorylation expression level of the p-FOXO1 protein. There were significant differences among the groups represented by different characters (*p* < 0.05). Abbreviations in the picture: normal control (C); model group (M); HFD + GABA-rich adzuki bean (TF3); and HFD + metformin group (TS).

As can be seen from Figure 9, the expression of the FOXO1 protein in the liver of mice in the M model group was higher than that in other groups, but there was no significant difference between the M model group and C normal control group. After 6 weeks of dietary and drug intervention, FOXO1 protein expression in the TF3 group and TS group was significantly down-regulated compared with that in the M group (*p* < 0.05), but there was no statistical significance between the TF3 group and M group (*p* > 0.05). In addition, the phosphorylation level of FOXO1 in the M model group was significantly higher than that in the C normal group (*p* < 0.05). After TF3 diet intervention, the phosphorylation level of FOXO1 protein in the M model group was increased by 20.95% compared with that in the M group, and the phosphorylation degree was similar to that of the TS group, suggesting that TF3 diet can promote FOXO1 protein phosphorylation.

## 4. Discussion

The beneficial effects of GABA on peripheral adipose islet β cells [17], skeletal muscle [18], lipid metabolism [19], and the brain [20] have been confirmed. Studies have shown that the GABA receptor (GABA_A_R) exists in the peripheral tissues of the liver and that GABA has a tissue-specific effect on the insulin pathway through GABA_A_R. In peripheral tissues, GABA can induce membrane depolarization in islet beta cells. In isolated rodent and human islets, GABA has been proven to activate AKT and promote the proliferation of beta cells and insulin release. GABA activates GABA_A_ receptors on beta cells by opening the voltage-gated calcium channel (VGCC) to induce depolarization and thus activate the PI3K/AKT pathway and insulin [21]. GABA has different effects on different islet cells through the GABAA receptor. It induces the hyperpolarization of alpha cells and the depolarization of beta cells and inhibits glucagon and insulin [22]. Therefore, PI3K/AKT may also be a potential target for the GABA-rich adzuki bean to regulate hepatic glucose metabolism.

Insulin resistance in the liver causes glucose metabolism disorder, which is another important feature of type 2 diabetes mellitus. The PI3K/AKT pathway is considered to be the most closely related pathway to insulin transduction [23]. Under normal circumstances, insulin will first bind to the receptor (INSR) on the cell surface. The activation of INSR tyrosine kinase triggers the cascade reaction of autophosphorylation and the substrate phosphorylation of INSR, which initiates the insulin pathway through the activation and recruitment of scaffold proteins [24]. IRS is expressed in hepatocytes with two subunits, IRS1 and IRS2, which act similarly in the liver. IRS1 can bind to the phosphorylated insulin receptor to play an insulin transduction role. IRS1 can then mediate hepatic glucose metabolism through PI3K and downstream AKT sites [25,26]. PI3K is an intracellular phosphatidylinositol kinase that once activated, promotes the phosphorylation of phosphatidylinositol 4, 5-diphosphate (PIP2) on the cell membrane at the D3 site of the inositol ring. The product of PIP3 can further bind to the pH site of AKT so that it stops at the pH site of inositol 3-phosphate-dependent protein kinase 1 (PDK1), which creates the necessary conditions for PDK1 to catalyze the phosphorylation of AKT [27]. AKT is the main component of the insulin downstream factor and the beginning of hepatic insulin conduction diversification, which will lead to a series of cascade reactions in the IRS1/PI3K/AKT pathway, in which GSK-3FOXO1mTORC1 and AMPK are the substrates of AKT, which can regulate glycogen synthesis, gluconeogenesis gene transcription, and related rate-limiting enzyme activity. These modulators synergistically control the large metabolic process of hepatic glucose synthesis and thus play an active role in insulin transduction and glucose metabolism [28].

When IR occurs in the liver, the tyrosine phosphorylation of IRS1 is severely damaged by hyperglycemia, leading to a decrease in the liver’s ability to absorb glucose, affecting proximal defects in the INSR, IRS1, PI3K, and AKT pathways and impairing the insulin transduction pathway. It appears that the activity of insulin hinders the synthesis of glycogen in the liver, and at the same time, the gluconeogenesis pathway affects the concentration of blood sugar in many respects, eventually causing metabolic diseases such as hyperglycemia and hyperlipidemia [29]. As mentioned before, FOXO1 is the target of AKT, and the main phosphorylation sites are Thr24, Ser256, and Ser319 [30]. On the one hand, FOXO1 can activate threonine phosphorylation under the regulation of the upstream PI3K/AKT pathway and trigger nuclear translocation, resulting in a decrease in the transcription ability of FOXO1 and thereby inhibiting the expression levels of *PEPCK* and *G6PC* genes in the liver. On the other hand, FOXO1 acts as a transcription factor that can tightly bind to the DNA target sequence in the promoter region of the gluconeogenesis rate-limiting enzyme and regulate the gene expression of important enzymes such as PEPCK and G-6-Pase in the gluconeogenesis pathway. It can be seen that FOXO1, GSK3β, AMPK, and other proteins are important regulatory factors in the insulin pathway. By increasing the phosphorylation expression levels of GSK3β and FOXO1 proteins, the activity of GSK3β and FOXO1 factors can be inhibited, thereby increasing the activity of GYS2 and inhibiting downstream gluconeogenesis. The activities of the key rate-limiting enzymes PEPCK and G-6-Pase enhance the ability of liver glycogen production and storage.

**Figure 10:**
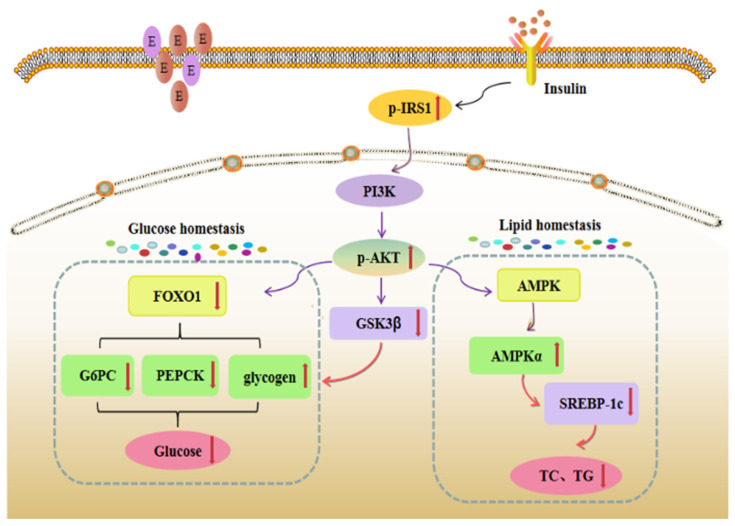
Liver insulin information pathway. The red upward arrow indicated that the activity of the gene, protein or enzyme in the study results is increased in the pathway, and the red downward arrow indicated that the activity of the gene, protein or enzyme in the study results is decreased in the pathway.

In this study, a high dose of the GABA-rich adzuki bean reduced the hepatic state of T2DM mice induced by HFD + STZ, promoted glycogen synthesis in the liver of T2DM mice, inhibited the activities of gluconeogenesis rate-limiting enzymes PEPCK and G-6-Pase in liver tissue, and significantly down-regulated the gene expression levels of *PEPCK*, *G6PC* and *FOXO1*, which are downstream factors of the insulin pathway. In addition, TF3 treatment increased the expression levels of AMPK α and IRS1-related proteins, activated AKT protease, and enhanced the expression level of AKT protein phosphorylation, which indicated that TF3 could regulate liver glucose metabolism in T2DM mice based on the IRS1/PI3K/AKT pathway. The specific transduction pathway is shown in Figure 10.

On the one hand, TF3 intervention can significantly increase the activity of IRS-1, which is the insulin receptor substrate in the liver tissue of T2DM mice, and then increase the level of AKT protein and AKT protein phosphorylation through a PI3K series cascade reaction, enhance insulin conduction ability, and relieve IR symptoms by improving insulin sensitivity. At the same time, the protein phosphorylation of GSK3 β downstream of AKT is enhanced, and the fluorescence intensity and gene expression level of GSK3β cells are down-regulated, which triggers the inhibition of glycogen synthase activity and the synthesis of glycogen and blood glucose [31]. On the other hand, TF3 dietary intervention can significantly down-regulate the gene expression level of *AKT* transcription factor *FOXO1* and up-regulate its protein phosphorylation level, thus inhibiting the transcription activity of FOXO1. It can also significantly down-regulate the gene expression of key gluconeogenesis enzymes PEPCK and G6PC in T2DM mice, inhibit the enzyme activities of PEPCK and G-6-Pase to weaken gluconeogenesis, and finally, improve glucose homeostasis in vivo. In addition, TF3 dietary intervention can also significantly up-regulate the gene expression of *AMPK α*, a key regulator of glucose and lipid metabolism, and down-regulate the expression of the *SREBP1c* gene to inhibit the synthesis of triglycerides and cholesterol in T2DM mice. Lipid accumulation in mice improved lipid metabolism disorder in vivo.

In conclusion, a GABA-rich adzuki bean diet can regulate the glucose metabolism of the T2DM mice liver through the IRS/PI3K/AKT-GSK, 3β,IRS/PI3K/AKT-FOXO1-PEPCK/G-6-Pase, and IRS/PI3K/AKT-AMPK α/SREBP1c pathways and has demonstrated potential in balancing blood glucose at liver tissue targets effectively. It is suggested that the TF3 diet may regulate blood glucose through multiple hepatic glucose metabolism pathways to alleviate T2DM glucose metabolism disorder.

## 5. Conclusions

In conclusion, a GABA-rich adzuki bean diet reduced the hepatic pathological state induced by HDF + STZ in T2DM mice, promoted glycogen synthesis in the liver of T2DM mice, inhibited the activities of gluconeogenesis rate-limiting enzymes PEPCK and G-6-Pase in the liver tissue, and significantly down-regulated the gene expression levels of *PEPCK*, *G6PC* and *FOXO1*, which are downstream factors of the insulin pathway. The adzuki bean, rich in GABA, can up-regulate the expression of the *AMPK α* gene and down-regulate the expression of the *SREBP1c* gene to inhibit the synthesis of triglycerides and cholesterol in T2DM mice. Derosa et al. [32] proved that GABA intervention could significantly improve the levels of TG and TC in HFD mice and speculated that this phenomenon might be related to the AMPK pathway. It can be concluded that the GABA-rich adzuki bean can promote hepatic glycogen synthesis and inhibit hepatic gluconeogenesis by regulating the expression of IRS/PI3K/AKT pathway genes and proteins.

## Figures and Tables

**Table 1:** Sequences of primers for genes.

Gene	Forward Primer	Reverse Primer	Amplification Length (bp)
*G6PC*	AGGTCGTGGCTGGAGTCTTGTC	AATGCAGGCGAAGCGGAATGG	359
*PEPCK*	AAGGAGTGGAGACCGCAGGAC	TGCCGAAGTTGTAGCCGAAGAAG	310
*FOXO1*	CCTGAGCCTGCTGGAGGAGAG	GCACGCTCTTCACCATCCACTC	353
*AMPKα*	GCAGAAGATTCGGAGCCTTGACG	GCATCAAGCAGGACGTTCTCAGG	300
*SREBP1c*	GCCATCGACTACATCCGCTTCTTG	AGTCACTACCACCACTGCTGCTAG	265
β-Actin	TCCAGCCTTCCTTCTTGGGTATG	CATCCTGTCAGCAATGCCTGGGTAC	155

## Data Availability

The original contributions presented in the study are included in the article, further inquiries can be directed to the corresponding author.
